# Impacts of Climate Change and Human Activity on the Habitat Distribution of *Metasequoia glyptostroboides*

**DOI:** 10.1002/ece3.71269

**Published:** 2025-04-10

**Authors:** Ming Li, Yu Sun, Yongsheng Yang, Xiujuan Zhang

**Affiliations:** ^1^ Key Laboratory of Adaptation and Evolution of Plateau Biota Northwest Institute of Plateau Biology, Chinese Academy of Sciences Xining China; ^2^ College of Horticulture and Gardening Yangtze University Jingzhou China

**Keywords:** Anthropocene, climate change, dispersal potential and limited, MigClim model, optimized MaxEnt model

## Abstract

Extensive evidence supports that global climate change influences shifts in species habitats due to alterations in hydrothermal conditions; however, neglecting dispersal capacities and limits significantly heightens uncertainties regarding spatial distribution patterns among different organisms. In this study, we compared the spatial distribution of *Metasequoia glyptostroboides* Hu & W.C. Cheng ( *M. glyptostroboides* ) in the current Anthropocene context to that in a climate‐only context, providing new insights into the effects of climate change, dispersal potential, and dispersal barriers on the habitat changes for *M. glyptostroboides* . By utilizing optimized MaxEnt and MigClim models, we predicted Mid‐Holocene (MH) conditions and potential colonizable habitats under three emission scenarios (SSP126, SSP245, and SSP585) for both the medium and long term. We also assessed habitat distribution and variation differences in future warm‐wet conditions and the Anthropocene context. The results revealed that (1) The Precipitation of driest month (BIO14), Mean diurnal range (Bio2) and human footprint (HFP) are the primary factors influencing the expansion or contraction of the habitats of *M. glyptostroboides* . Human footprint, farmland, roads, and construction land are the main contributors to habitat loss and fragmentation. (2) Habitats of *M. glyptostroboides* are expected to experience significant loss in the future. There is potential for recovery in South China under the SSP126 emission scenario, but human activities may hinder this recovery. Moderate human intervention is necessary in regions, such as Hubei, Hunan, Anhui, and Sichuan basins. (3) Due to human influence, the habitat and high‐suitability areas for *M. glyptostroboides* are projected to migrate northeastward. Under the SSP126 scenario, a trend of reverse migration may be observed in the long term. This study minimizes the uncertainty in predicting species distribution under climate change while providing theoretical support for future habitat conservation of *M. glyptostroboides* .

## Graphical abstract

Our research addresses the critical need to understand how climate change, combined with anthropogenic factors, influences the habitats of species. By employing advanced modeling techniques such as MaxEnt and MigClim, we have predicted the potential habitats of M. glyptostroboides during the mid‐Holocene and under three future emission scenarios (SSP126, SSP245, and SSP585). Our findings reveal that the The Precipitation of driest month (BIO14), Mean diurnal range (Bio2) and human footprint (HFP) are pivotal in determining habitat dynamics. Human activities, including infrastructure development, are identified as key drivers of habitat loss and fragmentation.

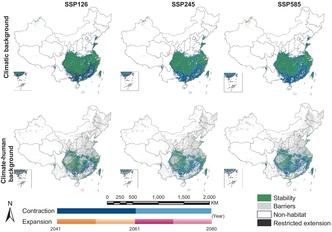

## 1 Introduction

Global climate change, driven by a confluence of factors, such as rapid industrialization, diverse human activities, and natural influences, represents one of the most formidable challenges we confront today, with global warming serving as its principal characteristic (Alexandre et al. 2021). The swift escalation of global warming engenders a myriad of issues on ecology, environment, and economics (David et al. 2017; Maximilian et al. 2024). Notably, the interplay between global warming and biodiversity has emerged as a focal point for research in recent years (Carsten et al. 2019). The detrimental and unpredictable effects of global warming on ecosystems and biodiversity, including habitat fragmentation, species extinction, and the proliferation of non‐native species, have been extensively documented (Tim et al. 2015). Prior studies indicate that the risk of species extinction is likely to intensify with rising future global temperatures (Gretta et al. 2017; Mark 2015). Consequently, forecasting the distribution of potentially suitable habitats for various species and their responses to climate change can furnish scientific guidance for planning future plant introductions and cultivation strategies as well as ecological conservation efforts aimed at mitigating accelerated species extinctions globally (Shi et al. 2023).

The maximum entropy model (MaxEnt), highlighting the exceptional performance evaluated by the receiver operating characteristic (ROC) curve and the Kappa coefficient, was often demonstrated to possess superior performance in identifying dominant environmental factors, quantitatively describing variables, and accurately simulating habitats (Morales and Fernández 2020). Furthermore, it continues to exhibit enhanced accuracy even with a limited number of samples (van André et al. 2016). The ENMeval algorithm reduces ecological homogeneity deviation by combining multiple parameters and calculating optimal parameters (Robert et al. 2014).

Natural dispersion is a fundamental process of ecosystem self‐restoration and succession, representing a universal phenomenon observed across nearly all living organisms (Ran 2006). In the absence of human intervention, like other sessile organisms, the natural dispersal of plants predominantly occurs passively; their seeds or loose spores are transported from parent plants by animals, wind, and water. However, it can also be active and propagated through intrinsic characteristics (Katelyn et al. 2024; Ran 2006). Natural dispersal events frequently enable species' geographic distributions to extend beyond native ranges into new potentially suitable habitats, exemplified by recolonization following ice ages and the transfer or expansion of species ranges (Dunja et al. 2021). Climate change and alterations in habitat conditions caused by humans further heighten the likelihood of adaptive changes; consequently, dispersal frequency increases as anthropogenic activities disrupt biogeographic barriers, and climate similarities will increasingly define biogeography in an era marked by global change while facilitating the breakdown of geographic dispersal barriers (César et al. 2015). As a result, species gradually occupy their potential environmental niches with individuals extending beyond ecological time constraints (Katelyn et al. 2024). Additionally, humans modify existing natural diffusion modes to directly influence these processes. For instance, constructing corridors to surmount biogeographic obstacles or utilizing man‐made plastic rafts for passive diffusion (James et al. 2018). Consequently, it is imperative to consider the changes in species niches induced by human activities and natural dispersion (Katelyn et al. 2024).

The potential colonizable habitats, the MigClim model has been introduced to address the limitations of species distribution modeling (Robert et al. 2014; Robin and Guisan 2009; Zhou et al. 2023). The potential colonizable habitat considers factors, such as limited seed dispersal distance, time to reach reproductive maturity, suitable habitat gaps, or dispersal barriers. The MigClim model is highly compatible and flexible for identifying existing species distribution modeling software. It projects species‐specific dispersal limits based on potentially suitable habitats and human land use status to accurately reflect habitat fragmentation and future changes (Robin et al. 2012). The integration of MaxEnt and MigClim models offers a powerful tool for forecasting future habitat distribution, fragmentation, and migration patterns for species. This has important implications for biodiversity conservation and reserve planning efforts. By leveraging these advanced models, researchers and conservationists can better anticipate how species will respond to changing environmental conditions, informing proactive conservation strategies and ensuring the long‐term survival of valuable ecosystems.

*Metasequoia glyptostroboides* Hu & W.C. Cheng ( *M. glyptostroboides* ), commonly referred to as the “living fossil,” is a rare and remarkable remnant plant species globally, representing the sole surviving in the Cupressaceae family (Li et al. 2012). This monoecious plant undergoes cross‐pollination via wind pollination, where pollen is dispersed by air currents. Currently, the wild population of Chinese metasequoia is naturally confined to an area at the confluence of Hubei, Hunan, and Chongqing provinces, encompassing approximately 800 km^2^. The region features a horseshoe‐shaped terrain surrounded by mountains on three sides and lies within geographical coordinates ranging from 108°20′ to 109°30′ east longitude and 29°25′ to 30°10′ north latitude (Li et al. 2012); it falls under a subtropical monsoon climate characterized by abundant rainfall distributed evenly throughout the year. Furthermore, *M. glyptostroboides* is classified as endangered on the IUCN Red List, biological resource use, agriculture, and aquaculture are primary threats contributing to its endangerment (https://doi.org/10.2305/IUCN.UK.2013‐1.RLTS.T32317A2814244.en, accessed on 08 March 2025). In the future, climate change and habitat fragmentation are likely to be significant contributors to its decline (Hamrick 2004; Linda et al. 2011). Research indicates that during the Cretaceous period (~ 100 million years ago), *M. glyptostroboides* co‐dominated ancient wetland forests alongside *Glyptostrobus pensilis*; both exhibit similar ecological traits predominantly found in native habitats with moderate disturbance mechanisms and minimal competition (Cindy et al. 2019). In recent decades, human activities, such as logging, seed trading, and planting economic crops beneath forest canopies have led to extensive deforestation within the habitats for *M. glyptostroboides* . This has directly diminished species richness in these areas while adversely affecting population growth rates for *M. glyptostroboides* itself. If current trends persist without intervention, it will be increasingly challenging for *M. glyptostroboides* to sustain its natural range (Cindy et al. 2011). However, previous research has primarily concentrated on physiological characteristics and genetic diversity within populations of *M. glyptostroboides* ; there remains a paucity of studies predicting potentially suitable growth areas for this species (Chen et al. 2011; Dong et al. 2011). Consequently, forecasting spatial patterns of potential distribution using SDMs, and analyzing critical environmental factors influencing geographic distribution, holds significant implications for conservation efforts aimed at cultivating or restoring the populations of *M. glyptostroboides* . The substantial impact of climatic factors as determinants for species distributions has been widely acknowledged; these elements intricately shape niche preferences and habitat suitability ultimately defining geographic boundaries for various taxa (He et al. 2023; Mehmet et al. 2023). From 1960 through 2016, regional climate data indicate a warming trend that poses risks: should temperatures rise further coupled with intensified drought conditions, the moisture‐dependent nature of *M. glyptostroboides* may face dire consequences threatening its survival prospects, it may largely in part due to weak natural regeneration capabilities (Cindy et al. 2011; Zhao et al. 2020). Furthermore, evidenced by limited natural regeneration observed within primary forest settings necessitates urgent investigation into how future climatic shifts might alter existing habitats thereby informing effective strategies designed mitigate accelerated extinction risk.

In this study, we aimed to assess the future habitat changes of *M. glyptostroboides* in the Anthropocene and to compare the impacts of both human and natural factors on its spatial distribution. We employed the optimized MaxEnt and Migclim models to analyze habitat degradation and potential shifts in suitable habitats for *M. glyptostroboides* within the context of current climatic conditions and anthropogenic activities. Additionally, we projected potential suitable areas from geological history (Mid‐Holence) through current suitability changes, as well as future medium to long term under the low‐emission scenarios (SSP126), intermediate emission pathways (SSP245) and high‐emission trajectories (SSP585). The response of *M. glyptostroboides* to warming and increased humidity under climate change, along with the influence of human activities on its habitat alterations, provides a practical reference for resource conservation, introduction, cultivation, and rational utilization. The specific objectives of this study included: (a) Identifying key driving forces influencing changes in the distribution of *M. glyptostroboides*; (b) comparing its distribution across climatic contexts versus those shaped by anthropogenic influences; (c) determining future migration patterns within suitable and highly suitable areas for *M. glyptostroboides*.

## 2 Materials and Methods

### 2.1 Collection and Screening for Species Occurrence Data

**FIGURE 1:**
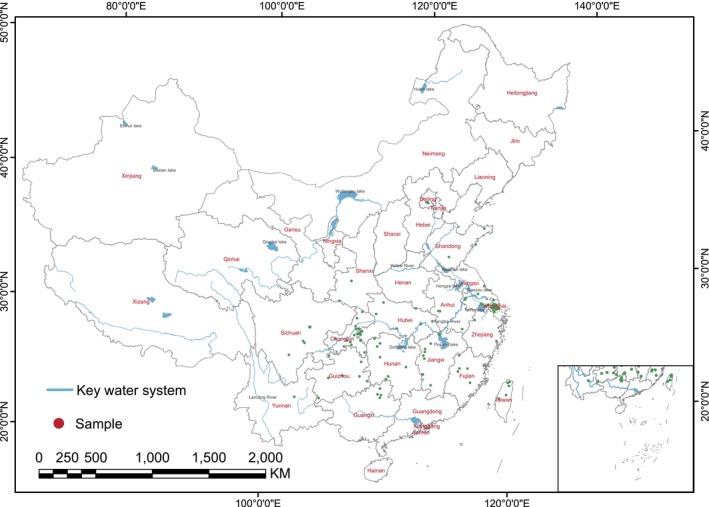
Distribution of *M. glyptostroboides* used in this study.

In this study, we obtained the occurrence data of *M. glyptostroboides* from the Global Biodiversity Information Facility (GBIF, http://www.gbif.org/, accessed on 20 August 2024) and the Chinese Virtual Herbarium (CVH, http://www.cvh.ac.cn, accessed on 20 August 2024). We selected and screened the recorded species sample sites from the GBIF within the Chinese scope, which included continental China, Macao China, Hongkong China, and China Taipei, deleting data with no images and coordinates. We utilized the coordinate picker in the Baidu map to collect specimen record data in CVH and supplemented it with literature sampling data (Yang et al. 2017), resulting in a total of 142 distributed samples. However, the presence of dense point sets may lead to overfitting and exaggeration of digital model simulation results. Initially, EnmTools software was employed to construct a 5 × 5 km spatial grid, removing duplicate values and conducting redundant screening based on the principle of retaining one sample point within each grid (Li et al. 2023). Subsequently, the geosphere package (R4.3.3) was used to calculate the distance between point sets. A 5 × 5 km buffer was established for secondary screening (Li et al. 2024), yielding 120 valid samples compiled and converted into CSV format files for habitat modeling (Figure 1).

### 2.2 Environmental Variables Information and Data Processing

Considering the variability of habitat conditions and the uncertainty of impact factors, we chose typical abiotic factors (climate, topography, and soil) and human activity elements (human footprint) related to *M. glyptostroboides* distribution. A total of 57 environmental variables were selected for model construction. Climate data, including Mid‐Holocene (MH), current and future climates were all achieved from the WorldClim Global Climate Database (https://www.worldclim.org/, accessed on 20 August 2024) with a resolution of 2.5 arcminutes (ca. 5 km). The current climate environment variables were utilized as part of the factors in constructing the initial model, composed of 19 bioclimatic variables, and its period was 1970–2000. Mid‐Holocene (MH, 6000 years. B.P) was generated by Community Climate System Model version 4 (CCSM4) developed by the National Center for Atmospheric Research (NCAR), which exhibited a high level of accuracy in simulating and predicting climate variables (Bette et al. 2006). The time period for the future climate data ranged from 2041 to 2080 and was divided into two time periods for 20‐year intervals, engendered by the average of the three global climate models (GCMs, including BCC‐CSM2‐MR, EC‐EARTH3‐veg and MPI‐ ESM1‐2‐HR), which can be used to reduce the biases from the calibration and data recording biases among the models. These models were included in the IPCC sixth assessment report (AR6) and showed higher precision in simulating the future climate (Moetasim et al. 2022). Three scenarios under the SSPs (Shared Socio‐economic Pathways) were selected as follows: SSP126 (sustainability pathway), SSP245 (medium emission pathway), and SSP585 (fossil fuel pathway). These scenarios could reflect the best and worst climate change conditions in the future (IPCC 2022). Topographic variables, including elevation, which was obtained from the WorldClim website with a resolution of 30 arcseconds (ca. 1 km), slope, and aspect, were derived using surface analysis tools in ArcGIS 10.8. Soil data were obtained from the Harmonized World Soil Database (HWSD, www.fao.org/, accessed on 20 August 2024) with a resolution of 30 arcseconds, resampled into 2.5 arcminutes above to ensure data consistency (Meng and Wang 2018). The Human footprint (HFP), sourced from the Socioeconomic Data and Applications Center (SEDAC, http://sedac.ciesin.columbia.edu, accessed on 20 August 2024), represents a data layer utilizing the human impact index to objectively and comprehensively depict the intensity and spatial distribution of human activities (Oscar et al. 2016).

### 2.3 Correction Analysis and Data Screening

**FIGURE 2:**
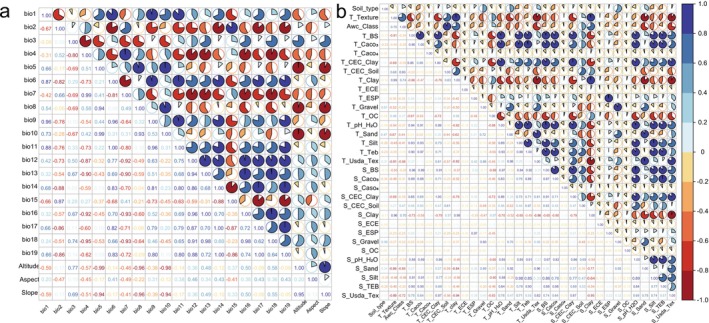
Correlation heatmap of environmental variables for *M. glyptostroboides*. Brunet colors indicate a stronger correlation or collinearity strength for two factors: (a) Correlation for climatic factors and (b) Correlation for soil factors.

In this study, Pearson correlation analysis and a Jackknife test embedded in the model were utilized to identify significant environmental factors. Initially, environmental variables with a contribution rate exceeding 10% were retained based on the Jackknife assessment, while those with no contribution were excluded (Mariano et al. 2024). Subsequently, Pearson correlation analysis, calculated by the corrplot package in R, was employed to assess correlations among various factors (Li et al. 2024). If there exists an environmental variable exhibiting a high correlation, specifically with an absolute correlation coefficient |R| ≥ 0.7, the variable with the higher contribution rate should be selected (Profillidis and Botzoris 2019). Ultimately, 25 environmental factors were selected for model prediction, including 7 climate data (Bio1, Bio2, Bio3, Bio4, Bio14, Bio15, Bio18), 3 topographic variables (Altitude, Slope, Aspect), and 14 Soil variables (Soil type, T_texture, Awc_class, T_BS, T_CEC_Clay, T_ESP, T_OC, T_pH_H_2_O, T_Usda_Tex, S_clay, T_ESP, S_Gravel, S_Silt, S_TEB, S_Usda_Tex, and HFP) (Figure 2).

### 2.4 Construction, Optimization, and Evaluation of MaxEnt

Previous research has demonstrated that MaxEnt quantified the statistical relationship between species' distribution and predictor variables through a combined effect of regularization multiplier (RM) and feature combination (FC) (Dan and Stephanie 2011). Although regularization helped prevent overfitting, artificial spatial autocorrelations (such as sampling bias) between training and test data partitions may inflate the metrics used to assess model performance, resulting in poor model performance (Duccio et al. 2023; Robert et al. 2014). In this study, the Enmeval 2.0 R package was utilized to assess the optimal parameter settings for MaxEnt, aiming to minimize model omission rates and mitigate overfitting issues (Zhou et al. 2023). RM, adjusted the regularization level, ranging from 0.1 to 4.0 with 0.1 intervals. FC limited the types of transformational relationships in the modeling process, including L (Linear), LQ (Quadratic), LQH, H (Fragmentation), LQHP (Product), and LQHPT (Threshold). A total of 240 combinations were utilized to compute the optimal parameter combination. The difference between the training and testing AUC (diff. AUC, calculated as AUCtrain—AUCtest) and the value of 10% training omission rate (OR10) were applied to examine the model's fit relative to the distribution points of native species, whereas Delta.AICc was used to assess both model complexity and its fitting accuracy (Li et al. 2024; Robert et al. 2014). Akaike Information Criterion correction (AICc) served as a standard for assessing the goodness‐of‐fit of statistical models; it effectively balanced the complexity of the estimated model against how well the data were represented by that model. The AICc prioritizes models with lower values, thereby favoring those that achieve optimal parsimony (Joseph and Andrew 2011). The selected parameter combination emerges in Table 1.

**TABLE 1:** Maxent optimized parameter measured by ENMeval.

Type	FC	RM	Mean.AUC	Mean.OR10	Avg. diff. AUC	Delta.AICc
Default	LQPH	1	0.9526	0.260	0.0256	583.67
Optimized	LQH	3.9	0.9559	0.098	0.0251	0

Abbreviations: Avg. diff. AUC, Average difference between the training and testing AUC; H, Fragmentation; L, Linear; Mean; Mean. OR10, Mean value of 10% training omission rate; P, Product; Q, Quadratic; T, Threshold.

To comprehend the influence of human activities and climate change on the suitable area of *M. glyptostroboides* , the MaxEnt 3.4.4 software (Princeton University Press, Princeton, NJ, USA) was utilized to construct two models. In the initial model, species distribution data and environmental factors (including climate, topography, and soil) were integrated into the sample and environmental layers of the model, respectively. Historical (Mid‐Holocene) and future environmental variables were incorporated into the projection layer to simulate the suitable habitats of *M. glyptostroboides* in the past, present, and future, within a climatic context, thereby elucidating the distribution of suitable areas under warm and dry adaptation (climatic characteristics of Mid‐Holocene). The second model integrated Human Footprint (HFP) data with environmental factors to evaluate potentially suitable areas influenced by the interaction of climate and human activities, projecting only current and future scenarios to understand the potential gains and losses of suitable areas of *M. glyptostroboides* due to anthropogenic impacts. The fundamental parameters of the model were established as follows: (1) Picked “Create response curves” and “Do jackknife to measure variable importance” to examine the relationship between species responses and environmental factors while identifying key driving forces (Phillips and Dudík 2008); select options such as “Make pictures of predictions”, “Write plot data”, and “Write background predictions” for producing statistical images and the graph datasets for ROC redrawing (Cao et al. 2020; Li et al. 2024). (2) Adjusted output settings to “logistic” which maintained the identical parameter configurations as MaxEnt, and offered more applicability and compatibility (Phillips and Dudík 2008); The “random seed” ensured reproducibility and verifiability of experimental results (Clayton et al. 2023). (3) 10 replicates for each simulation run of “cross‐validate” type were realized to build the model, it allowed for straightforward appraisal for model transferability, whereas 75% of the dataset was randomly selected as the training set, and the remaining 25% was used as testing set to minimize errors in both the training and test data (Low et al. 2021; Wang et al. 2024). We assessed the accuracy of models using the area under the receiver operating characteristic (ROC) analysis. The AUC with high values indicated a more accurate model. Model performance was classified as fail (0.5–0.6), bad (0.6–0.7), normal (0.7–0.8), good (0.8–0.9), and excellent (0.9–1) (Li et al. 2024; Wang et al. 2024).

To accurately and intuitively assess habitat suitability, it was essential to define parameters for suitability grades that delineate suitable habitats (Wang et al. 2019b). The model output was a continuous Habitat Suitability Index (HSI) map with a threshold range from 0 to 1. We employed the optimal assessment threshold (Maximum test sensitivity plus specificity threshold, MTSPS = 0.118) selected by MaxEnt to distinguish between suitable and unsuitable areas, categorizing suitable regions into four levels based on fixed value classifications: not suitable area (value≤MTSPS), generally suitable area (MTSPS < value ≤ 0.4), moderately suitable area (0.4 < value ≤ 0.6), and highly suitable area (0.6 < value) (Li et al. 2024; Shi et al. 2023). Additionally, we utilized ArcGIS “Extended Toolkit” (SDMtool box) to calculate changes in appropriate areas during the MH period, classifying these changes into three types: expansion, contraction, and stability; this toolkit was also employed to simulate the centroid and migration paths of both suitable and highly suitable areas under various scenarios while analyzing trends in species habitat change.

### 2.5 Construction of MigClim Model

The migration‐based simulation was conducted using the MigClim R package, elucidating the dispersal capacity and constraints of species through the reconstruction of dispersal routes and augmentation of dispersal barriers, providing a more accurate depiction of the future spatial distribution of species (Robin et al. 2012; Wang et al. 2019b). Dispersal capacity even played a crucial role in shaping the structure and dynamic changes of plant populations and communities (Ran et al. 2011), and the formula of dispersal simulation in MigClim was as follows:

$$P_{\mathrm{col}}=1-\prod_{i=1}^{n}\left(1-P_{\mathrm{Dis}p_{i}}\times P_{\mathrm{Pro}p_{i}}\right)\times P_{\mathrm{Inv}}$$

where *P*_*Dispi*_ is a probability function of the distance between the target cell and source cell I (values of *P*_*Disp*_ are entered through the [dispKernel] parameter) and reflects the fact that colonization probability decreases over distance. *P*_*Propi*_ is a probability that represents the propagule production potential of source cell I over time (values of *P*_*Prop*_ are entered via the [propaguleProd] parameter). *P*_*Disp*_ and *P*_*Prop*_ are implemented as discrete functions and can easily be modified to fit any shape of the seed dispersal curve and increase reproductive potential over time. *P*_*Inv*_ represents the habitat invasibility of the target cell and generally depends on its suitability for the species.

In this study, we delineated the appropriate areas for each period and assigned them a value of 1000, utilizing the MigClim model to compute the spatial distribution changes of *M. glyptostroboides* . Given that the recognition value of MigClim is 1000 times greater than that of MaxEnt, the potential suitability map was scaled accordingly to ensure seamless integration between the two models. Subsequently, the suitability threshold for the MigClim model was established at 118 (MTSPS*1000), whereas the current suitability area simulated by MaxEnt was designated as the initial distribution (IniDist). The future suitability areas predicted by the model outputs were defined as the habitat maps for 2050 (hsMap1) and 2070 (hsMap2). The environmental change steps (envChgSteps) were set to 2 to align with the appropriate zones for the subsequent periods (2050s and 2070s). Additionally, the number of dispersal steps (distSteps) was set to 20, corresponding to the number of diffusion events in the respective areas during each cycle. “[dispSteps][envChgSteps]” simulated the dynamic change of species' suitable areas from 2041 to 2080. We used dispersal kernel (dispkernel, a function that indicates the probability of a propagule to disperse at a given distance from its source) and propagule production potential (propaguleProd) to quantify the dispersal capacity in terms of biological characteristics and followed the negative exponential distribution (Ran 2006), ran the model 10 times to reduce error. Dispkernel was a function and entered as follows:

- “DispKernel=c(1.0, 0.4, 0.16, 0.06, 0.03)” (Robin and Guisan 2009).

We also utilized “strong” and “weak” proliferation barriers to effectively assess and quantify the impact of human activities on proliferation dynamics (Robin et al. 2012; Wang et al. 2019b). In this study, we set the “barrier style” in MigClim as strong to model the habitats under the sole climate scenarios and adjusted it as weak to strengthen the diffusivity of species under the climate and human joint effects (César et al. 2015). Additionally, this study extracted farmland, urban land, and transportation land as dispersal barriers from land use data obtained from GIMCloud (http://www.dsac.cn/, accessed on 30 March 2024), and we set up dispersal barriers for the habitat distribution *of M. glyptostroboides* in simulated climate‐human context experiments. The simulation results of the Migclim model included the initial distribution area (IniDist), increase, and contraction. The IniDist represented the area excluding the diffusion barriers in the region. Increase referred to the successfully colonized area during simulation; Loss indicates areas lost due to adverse habitat (Robin and Guisan 2009), then we used ArcGis 10.8 to visualize them.

## 3 Results

### 3.1 Predicted the Past and Current Potential Suitable Distribution Under Climatic Scenarios

**FIGURE 3:**
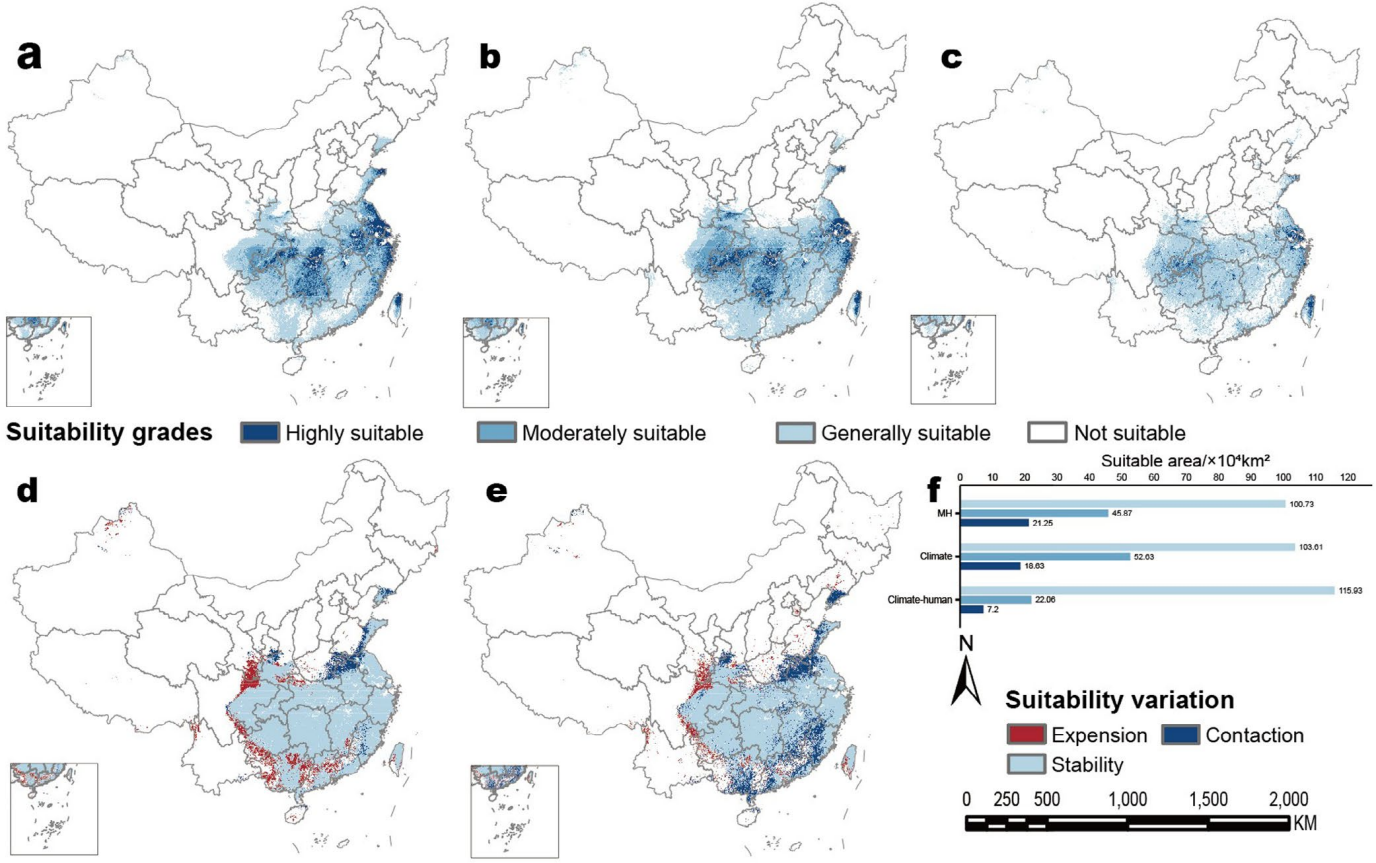
Spatial distribution and change of potentially suitable area for *M. glyptostroboides* in MH‐current period: (a) Suitable area of *M. glyptostroboides* in MH period, (b) current suitable area of *M. glyptostroboides* under the background of climate change, (c) current suitable area of *M. glyptostroboides* under the background of climate and humans, (d) spatial variation in a suitable area of *M. glyptostroboides* from MH period to the current under the background of climate change, (e) spatial variation in a suitable area of *M. glyptostroboides* from MH period to the current under the background of climate and humans, and (f) area of suitability grade for *M. glyptostroboides* .

Our SDMs indicated that the suitable habitats for *M. glyptostroboides* were predominantly located in Eastern and Central China (15°‐35° N, 100°‐130° E), with particularly high suitability observed in the Yangtze River basin and along the southeastern coast (past‐current). However, human activities have diminished this suitability level, consequently reducing the overall suitable habitat (Figure 3, Table 2). From the Mid‐Holocene to the current, suitable areas of *M. glyptostroboides* have expanded westward by 8.58%–12.63%, while northern and southern regions have experienced a loss of 7.64%–23.02% in suitable areas; conversely, there has been an increase in suitable area within Central Taiwan. Under current climatic conditions, the total suitable area has increased by 4.99%, indicating a trend where medium and low‐suitability areas are expanding while high‐suitability zones are declining. Notably, high‐suitability regions situated in southern Anhui and along the Hunan‐Hubei border have transitioned into medium‐suitability areas, whereas suitability levels around Chongqing and its bordering region with Hubei have improved. Under the climate‐human interactions influence, there has been an overall reduction of 14.44% in the suitable area of *M. glyptostroboides* ; moderate to high‐suitability areas have shifted to general‐suitability classifications, with certain coastal urban regions transitioning into non‐suitable categories.

**TABLE 2:** Change in current suitable area of *M. glyptostroboides* under different scenarios compared with MH period.

Scenarios	Area change/×10^4^/km^2^			Change rate/%			
	Increase	Reserved	Contraction	Increase	Reserved	Contraction	Change
MH ~ Climate	21.2	167.56	12.83	12.63	99.83	7.64	4.99
MH ~ Climate‐human	14.41	141.56	38.64	8.58	84.34	23.02	−14.44

### 3.2 Accuracy of the Simulated Prediction and Reactions of *M. glyptostroboides* to Climate Change and Anthropogenic Disturbances

**FIGURE 4:**
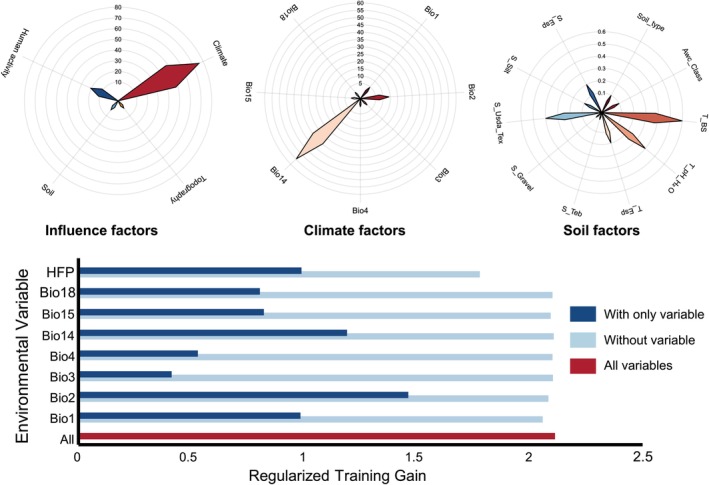
Contribution rate and regularized training gain to filter the dominant environmental variables for *M. glyptostroboides*.

We observed the optimized MaxEnt simulation was more effective for predictive modeling. The Enmeval optimization evaluation index indicated that, in comparison with the default parameters of the model, the average AUC value was increased by 0.35%, alongside a 62.31% reduction in the average omission rate, with diminished discrepancies between the training and test sets. Following the determination of optimal parameter configurations, we conducted simulation predictions which consistently yielded modeling AUC values exceeding 0.9, signifying robust model performance and enhanced reliability and accuracy in predictions (Figure 4). The research identified key environmental variables influencing the distribution of *M. glyptostroboides* through contribution rates, permutation importance, and regularization training gain values. Initially, we analyzed critical factors affecting the distribution of *M. glyptostroboides* using contribution rates; subsequently, we further examined these factors by integrating three indicators to ascertain dominant environmental variables impacting the distribution of *M. glyptostroboides* . Our results indicated that climate (75.7%) and human activities (20.5%) were pivotal drivers for *M. glyptostroboides* ; specifically, Bio2, Bio14, and HFP emerged as dominant environmental variables. Our findings found thatBio14 exhibited the highest contribution rate while univariate analysis revealed Bio2 had the greatest regularization gain value and higher permutation importance; notably, excluding HFP resulted in minimal model gains. Additionally, terrain (0.9%) and soil (2.9%) were recognized as significant contributors to *M. glyptostroboides* (Figure 4, Table 3).

**TABLE 3:** Contribution rate of environmental variables by MaxEnt for *M. glyptostroboides* .

Variables	Percent contribution (%)	Permutation iportance (%)	Variables	Percent contribution (%)	Permutation importance (%)
Bio14	54.7	0.2	S_Gravel	0.4	0.6
HFP	20.5	10.2	S_Usda_Tex	0.4	0.1
Bio2	14.2	58.5	T_Esp	0.2	0.7
Bio1	4.6	7.3	S_Esp	0.2	0.2
Bio3	0.9	2.5	Bio18	0.2	1.7
Bio15	0.9	4.7	Bio4	0.2	6.4
Slope	0.8	0.5	Altitude	0.1	2.6
T_BS	0.6	0.3	Soil_type	0.1	0.2
S_Teb	0.4	0.8	S_Silt	0.1	0
T_pH_H_2_O	0.4	1.7	Awc_class	0.1	0

**FIGURE 5:**
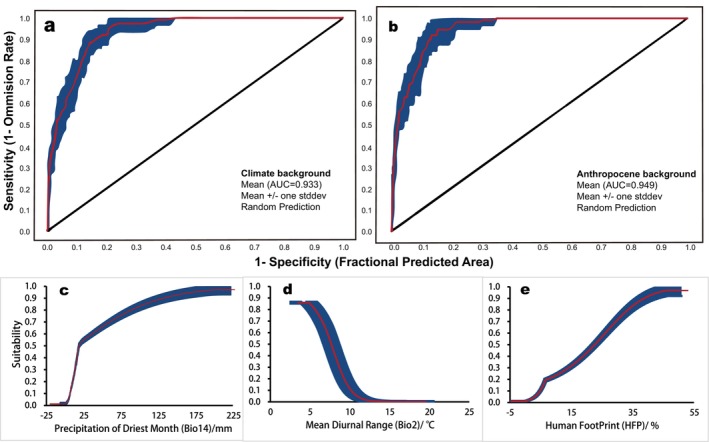
ROC curve and AUC validation for MaxEnt, and dominant environmental variables of response curves for *M. glyptostroboides* : (a) ROC and AUC under climate scenario; (b) ROC and AUC under climate‐human activities scenario; (c) precipitation of driest month; (d) mean diurnal range; (e) human footprint.

We further constructed response curves for dominant environmental variables to delineate suitable conditions for *M. glyptostroboides* growth. Our analysis demonstrated that *M. glyptostroboides* necessitates higher precipitation levels during its driest months and prefers stable temperature variations while exhibiting adaptability to regions characterized by elevated human activity indices (Figure 5c–e). Specifically, it was determined that the threshold of Bio14 above 18.58 mm represented an optimal range for *M. glyptostroboides* suitability, which increases proportionally with rising precipitation levels; furthermore, a diurnal temperature difference limit of 7.66°C was established beyond which suitability diminishes, and the smaller this temperature differential was observed to enhance overall suitability; additionally, *M. glyptostroboides* showed resilience toward areas with high human activity indices where its suitability escalates exponentially when HFP surpasses 22 .68%.

### 3.3 The Habitat Loss Attributable to Human Activities Significantly Surpasses the Effects of Climate Change

**FIGURE 6:**
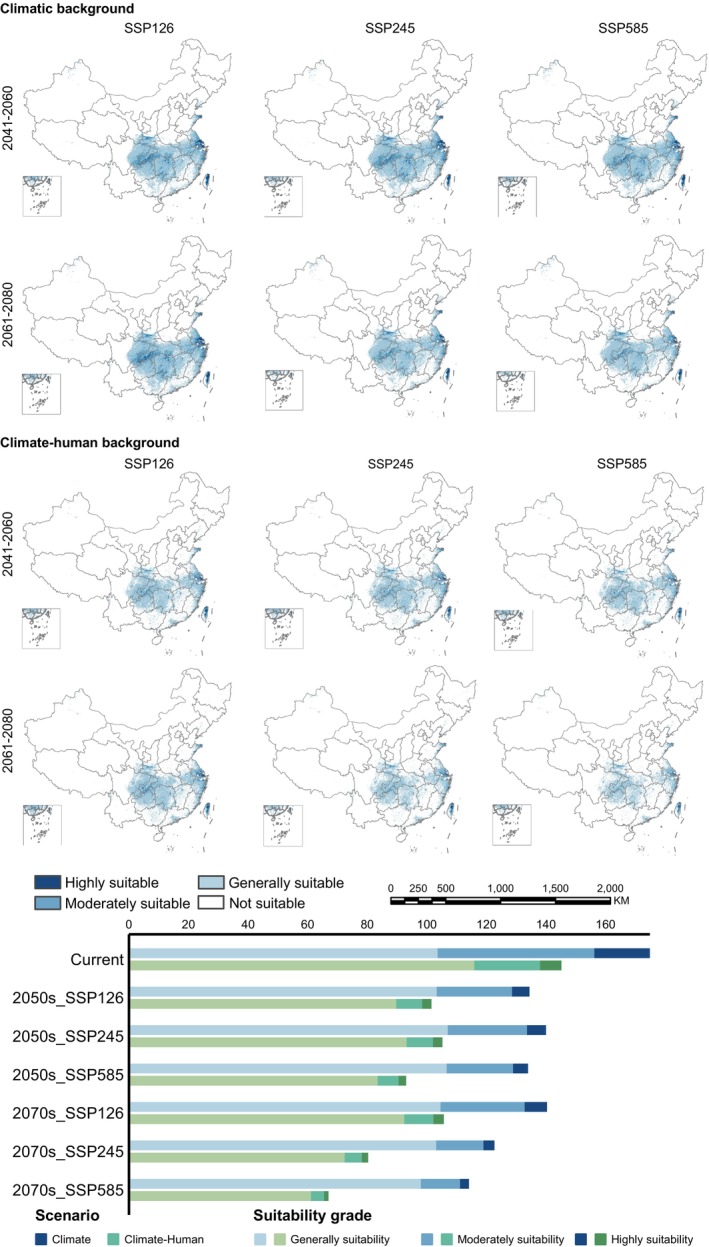
Distribution of potentially suitable areas of *M. glyptostroboides* under climatic scenarios in the future.

**FIGURE 7:**
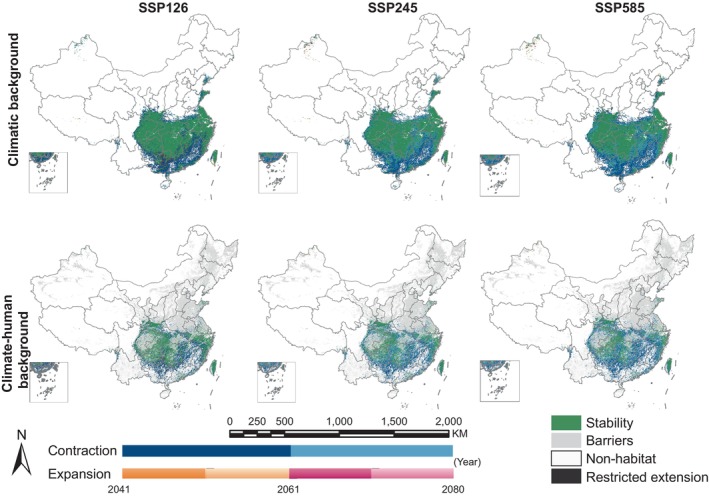
Dynamic change of potentially colonizable habitats for *M. glyptostroboides* under future climatic scenarios.

We employed MaxEnt and MigClim models to project the future habitat distribution of *M. glyptostroboides* under various emission scenarios influenced by both human activities and climate change (Figures 6 and 7, Table 4). Our findings indicated that the combined pressures from human activities and climate change result in a more severe habitat loss for *M. glyptostroboides* , projected to range between 17.63% and 54.46% in the future. Among the emission pathways, SSP126 exhibited the least habitat loss, whereas SSP585 was associated with the most pronounced degree of habitat loss and fragmentation. Stable habitats were primarily located in southeastern coastal regions as well as the border of Hubei, Chongqing, and Guizhou provinces. Considering climate change alone, habitats of *M. glyptostroboides* were expected to decline by 22.16%–34.11%, with mid‐term (2050s) projections indicating particularly acute losses during this period. The SSP245 pathway demonstrated minimal habitat loss (18.85%) compared with other scenarios, predominantly affecting the landscape of southern China; meanwhile, both SSP126 and SSP585 pathways will lead northern habitats to retreat southward. Under long‐term (2070s) conditions dictated by SSP126 emissions, habitats of *M. glyptostroboides* were anticipated to expand southward with gradual recovery observed in southern areas, resulting in a comparatively minor loss area (17.63%). Conversely, both SSP245 and SSP585 pathways will contribute further losses ranging from 10.17% to 11.69%, concentrated mainly within northern Anhui province along with Jiangxi and southeast Hunan provinces. Under scenarios combining climate change impacts alongside human activity influences, total projected habitat losses for *M. glyptostroboides* could reach between 28.13% and 54.46%. The medium‐term losses remain relatively low under the SSP245 pathway; however, long‐term projections suggested significant declines across Hubei, Jiangxi, and Guizhou regions over timeframes extending beyond this period. In contrast, trends associated with path SSP126 revealed substantial short‐term losses followed by potential recovery phases occurring later on toward southern territories; whereas continued degradation persists under scenario conditions defined by pathway SSP585, with mid‐term estimated reflecting a staggering cumulative reduction reaching up to ~ 37.76% coupled alongside an additional estimated decrease around 17.4% impacting southern portions throughout longer durations ahead. Significant habitat fragmentation would be expected to occur along the southeastern coastline and Central China, especially in Shanghai and Jiangsu, part was located in Anhui, Guizhou, and Hubei, as well as the Sichuan basin (Figure 7).

**TABLE 4:** Change of the suitable habitats for *M. glyptostroboides* under future climatic scenarios.

Climate scenarios/Anthropocene scenarios	Suitable area/×10^4^ km^2^	Suitable habitat/×10^4^ km^2^		Change rate /%			
		Area	Increase	Last	Increase	Last	Change
Current	174.88/145.18	170.28/88.98	—	—	—	—	—
2050s_SSP126	134.46/101.55	134.38/60.96	0.97/0.99	36.87/29.01	0.57/1.11	21.65/32.60	−21.08/−31.49
2050s_SSP245	139.99/105.23	139.88/63.19	1.70/1.28	32.1/27.07	1.00/1.43	18.85/30.42	−17.85/−28.99
2050s_SSP585	133.96/93.04	133.82/56.30	1.64/0.92	38.1/33.60	0.96/1.03	22.37/37.76	−21.41/−36.73
2070s_SSP126	140.34/105.72	140.25/63.95	7.71/4.45	37.74/29.48	4.53/5.00	22.16/33.13	−17.63/−28.13
2070s_SSP245	122.69/80.3	122.56/48.20	1.56/0.83	49.28/41.61	0.92/0.93	28.94/46.76	−28.02/−45.83
2070s_SSP585	114.14/66.99	113.92/40.52	1.72/0.62	58.08/49.08	1.01/0.70	34.11/55.16	−33.10/−54.46

### 3.4 Changes in Suitable Habitats and Migration Trend

**FIGURE 8:**
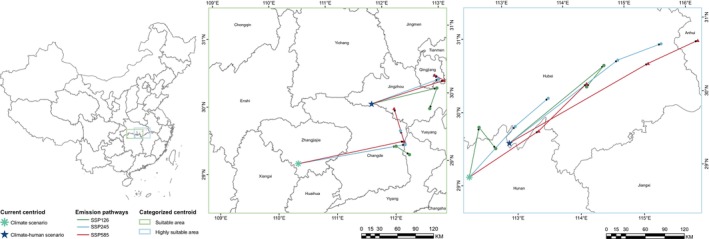
Centroid transfer in overall suitable areas and highly suitable areas for *Metasequoia glyptostroboides* under future different pathways.

We assessed the centroid and migration trajectory of both the overall suitable area and the highly suitable area for *M. glyptostroboides* under various future scenarios to evaluate how climate change and human activities influenced its suitability (Figure 8, Table 5). Our findings indicated that there were notable differences in the direction of centroid migration across suitable areas amidst anticipated climate changes. Under the SSP126 emission pathway, the suitable area was projected to migrate steadily northeastward, while a reverse migration trend was observed in the long term (2070s), and the highly suitable area showed the shortest migration distance, measuring only 60.02 km from its current centroid position. The potential suitable area changes for *M. glyptostroboides* under the SSP245 and SSP585 emission scenarios exhibited high consistency. However, the migration intensity was notably greater under the SSP585 scenario. Initially, the entire suitable area shifted northeastward, followed by a northwestward movement. Meanwhile, the highly suitable areas predominantly migrated northeastward. In terms of human activity impacts, it was found that the current centroid was situated in Jingzhou City; however, it will migrate northeast toward Qianjiang City over time. Notably, under SSP126 emissions, this process will reverse back toward but still in Jingzhou in the long term. Meanwhile, both overall and highly suitable areas exhibited their longest migrations (distance from the current centroid was 231.83 km and 332.57 km respectively) under SSP585 emissions. The mid‐term (2050s) represented a phase characterized by significant spatial alterations for *M. glyptostroboides* populations with peak migrations reaching up to 182.41 km recorded during this timeframe. Furthermore, our analysis revealed that high‐suitability zones within southwestern *M. glyptostroboides* were expected to transition northeastwards in future scenarios; this trend was more pronounced due to anthropogenic influences compared with those driven solely by climatic factors.

**TABLE 5:** Migration‐constrained change of the suitable habitats for *M. glyptostroboides* under future climatic scenarios.

Suitable area	Climate scenarios/Climate‐human scenarios	Longitude	Latitude	Migration distance compared with the previous period/km
Overall	Current	110°22′24″ E/111°42′31″ E	29°06′03″ N/29°55′17″ N	—
	2050s_SSP126	112°04′52″ E/112°51′54″ E	29°19′05″ N/30°04′58″ N	166.83/112.78
	2050s_SSP245	112°14′27″ E/112°54′24″ E	29°17′01″ N/30°12′41″ N	182.41/119.72
	2050s_SSP585	112°13′22″ E/112°59′56″ E	29°20′05″ N/30°11′19″ N	181.37/127.68
	2070s_SSP126	112°18′06″ E/112°42′44″ E	29°07′43″ N/29°47′14″ N	29.27/36.01
	2070s_SSP245	112°10′33″ E/113°38′05″ E	29°29′46″ N/30°06′21″ N	29.50/30.28
	2070s_SSP585	112°05′35″ E/112°50′25″ E	29°49′44″ N/30°16′34″ N	29.33/18.09
Highly	Current	112°16′08″ E/112°57′01″ E	29°06′41″ N/29°32′13″ N	—
	2050s_SSP126	112°29′01″ E/114°34′52″ E	29°47′01″ N/30°28′52″ N	77.58/188.89
	2050s_SSP245	113°03′57″ E/114°48′54″ E	29°45′45″ N/30°32′14″ N	105.55/211.17
	2050s_SSP585	113°26′10″ E/115°18′01″ E	29°40′07″ N/30°26′59″ N	128.96/248.02
	2070s_SSP126	112°43′32″ E/114°16′15″ E	29°28′33″ N/30°12′46″ N	41.43/42.16
	2070s_SSP245	113°38′05″ E/115°32′33″ E	30°06′21″ N/30°41′56″ N	67.03/30.70
	2070s_SSP585	114°16′27″ E/116°08′28″ E	30°15′24″ N/30°41′20″ N	103.89/84.79

*Note:* Overall indicates the potentially suitable area, defined as the region with a suitability score exceeding 0.118. Highly suitable areas are those with a suitability score greater than 0.6.

## 4 Discussion

Climate change and anthropogenic disturbances significantly influence the spatial distribution of trees globally (Chu et al. 2019; Liu et al. 2015). Here, we first disclose and compare the spatial distribution of *M. glyptostroboides* under the combined influences of climate change, land use practices, and intensity of human activity, confirming that both human activity intensity and land use patterns contribute to further habitat loss and fragmentation of *M. glyptostroboides* . Specifically, our findings reveal that under current climatic conditions, potential suitable areas for *M. glyptostroboides* amount to 174.88 × 10^4^ km^2^; however, this suitable area has diminished by 16.98% due to anthropogenic pressures alone, additionally exacerbated by current land use patterns which account for an extra 3.6% habitat loss (Figure 3, Table 2), indicating that climate change alone cannot adequately predict future shifts in the spatial distribution of *M. glyptostroboides* without considering multiple environmental variables beyond mere abiotic factors such as climate.

Abiotic conditions alongside dispersal capacity or mobility are critical determinants influencing species geographical distributions (Feng et al. 2024). Numerous studies have substantiated that hydrothermal conditions impact terrestrial plant distributions significantly; global warming is poised to alter forest ecosystem structures and functions markedly while prompting many endangered species' migrations toward higher latitudes or altitudes (Dai et al. 2016). Nevertheless, some investigations yield contradictory outcomes, they demonstrated a greater westward migration among trees compared with northward movements, highlighting that short‐term precipitation has more pronounced effects on trees relative to temperature fluctuations (Fei et al. 2017). Consistent with these, we analyzed how *M. glyptostroboides* responds to environmental variables; our findings indicate that temperature accounts for only 19.9% influence on its distribution whereas precipitation exerts a substantial weight at 55.8%, with terrain and soil contributing merely between 0.9% and 2.9% (Figure 4, Table 3). This underscores precipitation's pivotal role in facilitating plant habitat transitions while affirming its stronger impact alongside temperature on dynamic changes within the distribution of *M. glyptostroboides* compared with terrain or soil factors.

In addition, predicting species migration to new habitats often overlooks the critical factors of dispersal potential and limits (Karel et al. 2020). While species exhibit a more pronounced response to climate change compared with restriction factors, numerous studies frequently neglect the influence of geographic isolation and dispersal processes in modeling species habitats (Jonás et al. 2021). This oversight can lead to misestimations that significantly exceed actual environmental tolerances or realized niches for these species (Mariano et al. 2024). Similar to these, ample evidence suggests that species are likely to establish in areas characterized by suitable biotic interactions and abiotic conditions, enabling their spread over relevant timeframes (Wang et al. 2019a). In the Anthropocene, species, dispersion occurs at an accelerated rate, more frequently and across greater distances; alterations in land use practices may mitigate biogeographic isolation constraints, facilitating continuous connections between newly established distribution areas and original habitats that support habitat expansion (Katelyn et al. 2024). However, high levels of human interference coupled with natural habitat destruction may result in fragmentation issues for various species habitats (Guo et al. 2023; Weng et al. 2023). *Metasequoia glyptostroboides* Hu & W.C. Cheng, a unique relict tree endemic to China, is anticipated to be particularly vulnerable to both climate change and anthropogenic activities, potentially facing significant habitat loss in future scenarios.

The initial distribution patterns of many relict plants were shaped by paleoclimate scenarios primarily characterized by hydrothermal changes. The Holocene (11.7 thousand years ago) marked a pivotal period during which human activities began influencing natural environments substantially. Investigating the geographical distribution of *M. glyptostroboides* during this era provides valuable insights into the complexities and uncertainties surrounding spatial distribution changes from the current to the future (Jessica et al. 2020). Previous research has confirmed that during the late Holocene period, human activity exerted regulatory effects on regional vegetation within subtropical East Asia that may have surpassed those imposed by natural climatic variations, and this region usually serves as a refuge for several ancient relics (Liu et al. 2015). Furthermore, some researchers thought ancient endemic trees were distributed in southeastern China, where the hotspots were mostly warm and humid, concentrating in the climate‐stable area. In the future, precipitation in this area will be higher than that in the non‐hot spot area, and anthropogenic pressure will further impact the distribution pattern of endemic plants (Guo et al. 2023). In alignment with these, our findings reveal that the habitat of *M. glyptostroboides* expanded by 4.99% due solely to climatic conditions from the MH to the current period while experiencing a 14.44% reduction influenced by climate and anthropogenic disturbances. Furthermore, we anticipate medium‐term (2050s) losses ranging from 17.63% to 36.87%, compounded by long‐term (2070s) reductions driven largely by intensified human actions resulting in additional losses estimated between 16.54% and 17.73%. Notably under climatic backgrounds, the area deemed suitable remains consistent, but the addition of human activities increases their migration distances, and the SSP126 emission pathways in medium‐term reverse migrations suggest recovery possibilities for the habitats of *M. glyptostroboides* located in southwestern China (Figures 6 and 8, Table 5). At the same time, we observed severe habitat loss attributed directly to anthropogenic impacts along southeast coastal regions as well as central‐western territories; yet these lost areas could be recovered under SSP126 emission trajectories than under SSP585 pathways where fragmentation intensifies considerably across Shanghai and Jiangsu provinces compared with other locales examined herein (Figure 7, Table 4).

The parameter setting and extrapolation of SDMs can introduce uncertainty into the modeling results (Colin and Jack 2012; Emeric et al. 2014). It is crucial to carefully select appropriate statistical algorithms and parametric schemes based on the physiological and ecological characteristics of the target species and modeling data (Mariano et al. 2024). In this study, we utilize the ENMeval package to optimize MaxEnt and integrate the Migclim model to simulate species dispersal potential and limits, to predict the future distribution area of *M. glyptostroboides* . Following optimization, we observe a reduction in model omission rate by 62.31%, as well as an increase in Mean AUC by 0.35% (Table 1). These findings align with previous conclusions that the ENMeval algorithm effectively reduces model omission rates and complexity while improving accuracy (Cao et al. 2020). The AUC values by MaxEnt exceeded 0.9, indicating good reliability of our modeling efforts (Figure 5). The model extrapolation needs us to consider various factors for model extrapolation including the timeliness of distribution data, multiple environmental variables, and species diffusion potential (Li et al. 2024; Robert 2017). To address these considerations, we used species distribution data from 1970 onwards after conducting redundant screening to minimize sampling bias; analyzed multivariate environmental variables in contemporary contexts to account for spatiotemporal inconsistencies; simulated and calculated species dispersal capacity to explain the reasons for geographic isolation for habitats, and adjusted the strength of the diffusion barrier to accurately simulate the dynamic changes of *M. glyptostroboides* under both natural and human influences, all contributing toward improved predictive accuracy. However, it is crucial to recognize that the MaxEnt model is based solely on occurrence records. Although the model's strong simulation capability can mitigate the issue of incomplete coverage of suitable habitats due to limited occurrence records (Steven et al. 2004), bias in testing and the absence of inter‐biological relationships may still result in an overestimated simulated habitat range (Mariano et al. 2024). Therefore, future research should incorporate bia files to comprehensively reduce fitting errors and quantify biological interactions as diffusion constraints to more accurately reflect actual species distribution (Cao et al. 2020).

## 5 Conclusion

We meticulously and comprehensively assess the influences of global climate change, human activities, and species' dispersal capacity on the habitat transfer of *M. glyptostroboides* , comparing the difference in suitability for *M. glyptostroboides* under the independent climatic conditions and climate‐human joint conditions, which might be more intricate than the previous investigations of potential habitats. In particular, the future trend of habitat loss for *M. glyptostroboides* is projected to be more severe due to human activities than climate change. Southern coastal cities are expected to be the most affected. Although the SSP126 emission pathway shows some improvement, it also leads to significant changes in habitat conditions. Moreover, human activities are anticipated to drive the habitat of *M. glyptostroboides* to migrate northeastward, with high‐suitability areas shifting from central to eastern regions. The land use patterns are likely to cause habitat fragmentation, particularly in the eastern coastal areas and certain central regions, with Shanghai and Jiangsu experiencing more pronounced fragmentation than other cities. To address these challenges, it is crucial to enhance the protection and management of existing *M. glyptostroboides* resources in Shanghai and Jiangsu. Additionally, in light of the forecasted outcomes, it is recommended that moderate human interventions be implemented in the Hubei, Hunan, Anhui, and Sichuan basins. These interventions should focus on reducing agricultural and urban expansion to mitigate habitat loss. The objective is to connect fragmented habitats and protected areas, eliminate migration barriers, and assist *M. glyptostroboides* in adapting to the shifts in its distribution caused by rapid climate change.

## Author Contributions

**Ming Li:** formal analysis (equal), investigation (equal), methodology (equal), software (equal), validation (equal), writing – original draft (equal). **Yu Sun:** investigation (equal). **Yongsheng Yang:** funding acquisition (equal), project administration (equal), writing – review and editing (equal). **Xiujuan Zhang:** data curation (equal), funding acquisition (equal), project administration (equal), supervision (equal).

## Ethics Statement

All methods performed in this study were in compliance with the relevant institutional, national, and international guidelines and legislation.

## Consent

The authors have nothing to report.

## Conflicts of Interest

The authors declare no conflicts of interest.

## Supporting information


Data S1.

## Data Availability

The foundational data underpinning the conclusions of this study was sourced from the website data platform, which has been referenced in the article. The relevant code can be found in the Supporting Information.
