# Supplementary material for: Impacts of Climate Change and Human Activity on the Habitat Distribution of Metasequoia glyptostroboides
Source: Ecol Evol. 2025 Apr 10;15(4):e71269. doi: 10.1002/ece3.71269 (PMC11985169; doi:10.1002/ece3.71269)
Supplement: Supplementary file 1 — Data S1. [file ECE3-15-e71269-s001.docx]

R code for parameter optimization for Maxent model

setwd("D:\\")

library(rJava)

library(ENMeval)

library(raster)

library('ecospat')

system.file("java", package="dismo")

env.files <- list.files(path ="Environmental-data", pattern = ".tif", full.names =TRUE) #"Environmental-data"

env.files <-stack(env.files)

A<-read.csv("species.csv")

occ_<- A[,-1]

length(which(!is.na(values(subset(env.files,1)))))

enmeval_results <- ENMevaluate(occ_, env.files, partitions="block",n.bg=10000,

RMvalues =seq(0.1,4,0.1),

fc = c("L", "H", "LQ", "LQH", "LQHP", "LQHPT"), algorithm='maxent.jar')

options(java.parameters='-Xmx6144m')

gc()

memory.limit()

write.csv(enmeval_results@results, "enmeval_results.csv")

print(enmeval_results@results)

R code for Migclim model construction

setwd("D:\\ ")

library(migclim)

library(SDMTools)

library(raster)

data <- read.csv(" .csv",header=TRUE)

MigClim.migrate(iniDist=data[,1:3],hsMap=data[,4:7],

rcThreshold=200,barrier=data[,8],barrierType="strong",

envChgSteps=4,dispSteps=20,dispKernel=c(1.0,0.4,0.16,0.06,0.03),

iniMatAge=1,propaguleProd=c(0.01,0.08,0.5,0.92),lddFreq=0.01,

lddMinDist=6,lddMaxDist=15,simulName="MigClimTest",replicateNb=10,

overWrite=TRUE,testMode=FALSE,fullOutput=FALSE,keepTempFiles=FALSE)
